# Tuméfaction nasogénienne: ne pas oublier un kyste nasolabial

**DOI:** 10.11604/pamj.2017.26.202.11391

**Published:** 2017-04-13

**Authors:** Moncef Sellami, Abdelmonem Ghorbel

**Affiliations:** 1 Service d’'ORL et de Chirurgie Cervico-faciale du CHU Habib Bourguiba, Sfax, Tunisie

**Keywords:** Kyste nasolabial, non odontogène, obstruction nasale, Nasolabial cyst, non-odontogenic, nasal obstruction

## Image en médecine

Le kyste nasolabial ou kyste du seuil narinaire est une pathologie rare qui doit être évoquée devant toute masse kystique du vestibule nasal. Il s'agit d'une lésion kystique d'origine épithéliale, non odontogène à développement maxillaire extraosseux. Le diagnostic est fortement suspecté cliniquement et la taille du kyste peut atteindre 3 à 4 cm entrainant ainsi une déformation faciale. La tomodensitométrie conforte le diagnostic et évalue les rapports avec les structures adjacentes. Le traitement est chirurgical et s'associe un faible taux de récidive. La voie vestibulaire est l'bord privilégié qui permet l'exérèse totale du kyste. Nous rapportons le cas d'une femme âgée de 64 ans qui a consulté pour une tuméfaction nasogénienne gauche évoluant depuis 16 ans. Cette tuméfaction a augmenté progressivement de taille et s'est associée à une obstruction nasale gauche d'aggravation progressive. L'examen a montré une tuméfaction rénitente du sillon nasogénien gauche étendue en infra-orbitaire et au niveau du plancher narinaire gauche faisant 5 cm de grand axe. Une TDM du massif facial a montré une formation kystique du vestibule narinaire réalisant une large empreinte sur l'os maxillaire avoisinant. La patiente a été opérée par voie vestibulaire permettant l'exérèse d'un gros kyste très adhérant au plancher narinaire gauche et dont la dissection en haut s'est faite au voisinage du foramen infra-orbitaire. Les suites opératoires ont simples L'examen anatomopathologique a confirmé le diagnostic d'un kyste nasolabial. Aucune récidive n'a été notée après un recul de 3 ans.

**Figure 1:**
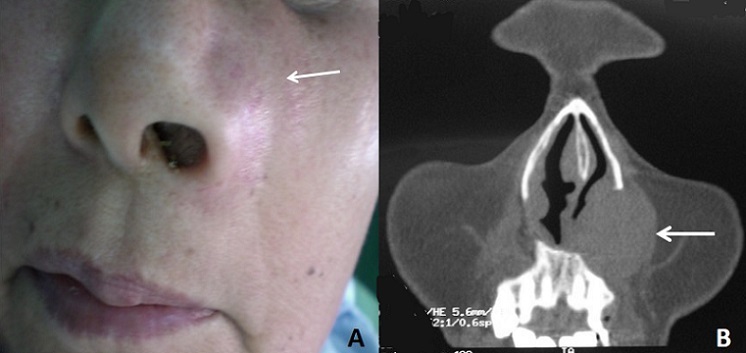
(A) tuméfaction rénitente du sillon nasogénien gauche étendue en infra-orbitaire et au niveau du plancher narinaire gauche faisant 5 cm de grand axe. La tomodensitométrie du massif facial en coupe coronale et coupe axiale; (B) montre une formation kystique du vestibule narinaire de 50 X 35 mm donnant une large empreinte sur l’os maxillaire avoisinant

